# Supplementary material for: Heat adaptation of phage T7 under an extended genetic code
Source: Virus Evol. 2021 Dec 1;7(2):veab100. doi: 10.1093/ve/veab100 (PMC8923235; doi:10.1093/ve/veab100)
Supplement: veab100_Supp [file veab100_supp.zip › Sequencing_Analysis.zip!/Sequencing_Analysis/A2/evidence/JC_54.html]

BRESEQ :: Evidence


| New junction evidence | | | | | | | | | | | |
| --- | --- | --- | --- | --- | --- | --- | --- | --- | --- | --- | --- |
|  | | seq id | position | reads (cov) | reads (cov) | score | skew | freq | annotation | gene | product |
| \* | ? | NC\_001604.1 | = 16476 | 362 (0.790) | 39 (0.090) | 31/590 | 11.1 | 9.2% | intergenic (–/–) | *–/–* | –/– |
|  | ? | NC\_001604.1 | 17143 = | 418 (0.930) |  |  |  |  | intergenic (–/–) | *–/–* | –/– |
| Rejected: Coverage evenness skew score above cutoff. | | | | | | | | | | | |
| Rejected: Frequency below/above cutoff threshold. | | | | | | | | | | | |

`CGCTGCCTTGAATACCCTACTGCAATCTGCTGGTGCTCTCATCTGCAAACTGTGGATTATCAAGACCGAAGAGATGCTCGTAGAGAAAGGCTTGAAGCATGGCTGGGATGGGGACTTTGCGTACATGGCATGGGTACATGATGAAATCCAAGTAGGCTGCCGTACCGAAGAGATTGCTCAGGTGGTCATTGAGACCGCACAAGAAGCGATGCGCTGGGTTGGAGACCACTGGAACTTCCGGTGTCTTCTGGATACCGAAGGTAAGATGGGTCCTAATTGGGCGATTTGCCACTGATACAGGAGG‑‑‑‑‑‑‑‑‑‑‑‑‑‑‑‑‑‑‑‑‑‑‑‑‑‑‑‑‑‑‑‑‑‑‑‑‑‑‑‑‑‑‑‑‑‑‑‑‑‑‑‑‑‑‑‑‑‑‑‑‑‑‑‑‑‑‑‑‑‑‑‑‑‑‑‑‑‑‑‑‑‑‑‑‑‑‑‑‑‑‑‑‑‑‑‑‑‑‑‑‑‑‑‑‑‑‑‑‑‑‑‑‑‑‑‑‑‑‑‑‑‑‑‑‑‑‑‑‑‑‑‑‑‑‑‑‑‑‑‑‑‑‑‑‑‑‑‑‑‑‑‑‑‑‑‑‑‑‑‑‑‑‑‑‑‑‑‑‑‑‑‑‑‑‑‑‑‑‑‑‑‑‑‑‑‑‑‑‑‑‑‑‑‑‑‑‑‑‑‑‑‑‑‑‑‑‑‑‑‑‑‑‑‑‑‑‑‑‑‑‑‑‑‑‑‑‑‑‑‑‑‑‑‑‑‑‑‑‑‑‑‑‑‑‑‑‑‑‑‑‑‑‑‑‑‑‑‑‑‑‑‑‑‑‑‑‑‑‑‑‑‑‑‑‑‑‑‑‑‑‑‑‑‑‑‑‑‑‑‑‑‑‑‑‑‑‑‑‑‑‑  >  NC_001604.1/16173‑16476`  
`‑‑‑‑‑‑‑‑‑‑‑‑‑‑‑‑‑‑‑‑‑‑‑‑‑‑‑‑‑‑‑‑‑‑‑‑‑‑‑‑‑‑‑‑‑‑‑‑‑‑‑‑‑‑‑‑‑‑‑‑‑‑‑‑‑‑‑‑‑‑‑‑‑‑‑‑‑‑‑‑‑‑‑‑‑‑‑‑‑‑‑‑‑‑‑‑‑‑‑‑‑‑‑‑‑‑‑‑‑‑‑‑‑‑‑‑‑‑‑‑‑‑‑‑‑‑‑‑‑‑‑‑‑‑‑‑‑‑‑‑‑‑‑‑‑‑‑‑‑‑‑‑‑‑‑‑‑‑‑‑‑‑‑‑‑‑‑‑‑‑‑‑‑‑‑‑‑‑‑‑‑‑‑‑‑‑‑‑‑‑‑‑‑‑‑‑‑‑‑‑‑‑‑‑‑‑‑‑‑‑‑‑‑‑‑‑‑‑‑‑‑‑‑‑‑‑‑‑‑‑‑‑‑‑‑‑‑‑‑‑‑‑‑‑‑‑‑‑‑‑‑‑‑‑‑‑‑‑‑‑‑‑‑‑‑‑‑‑‑‑‑‑‑‑‑‑‑‑‑‑‑‑‑‑‑‑‑‑‑‑‑‑‑‑‑‑‑‑‑ggaggTGTTCTGA‑TGTCTGACTACCTGAAAGTGCTGCAAGCAATCAAAAGTTGCCCTAAGACTTTCCAGTCCAACTATGTACGGAACAATGCGAGCCTCGTAGCGGAGGCCGCTTCCCGTGGTCACATCTCGTGCCTGACTACTAGTGGACGTAACGGTGGCGCTTGGGAAATCACTGCTTCCGGTACTCGCTTTCTGAAACGAATGGGAGGATGTGTCTAATGTCTCGTGACCTTGTGACTATTCCACGCGATGTGTGGAACGATATACAGGGCTACATCGACTCTCTGGAACGTGAGAACGAT  >  NC_001604.1/17143‑17442`  

                                                                                                                                                                                                                                                                                                                                                                                                                                                                                                                                                                                                                               
`gatgtgtataagagacagACTGCAATCTGCTGGTGCTCTCATCTGCAAACTGTGGATTATAAAGACCGAAGAGATGCTAGTAGAGAAAGGCTTGAAGCATGGCTGGGATGGGGACTTTGCGTACATGGCATGGGTACATGATGAAATCCAAGTAGGCTGCCGTACCGAAGAGATTGCTCAGGTGGTCATTGAGACCGCACAAGAAGCGATGCGCTGGGTTGGAGACCACTGGAACTTCCGGTGTCTTCTGGATACCGAAGGTAAGATGGGTCCTAATTGGGCGATTTGCCACTGATACAGG                                                                                                                                                                                                                                                                                                                  <  1:11504/283‑1`  
`ctcggagatgtgtataagatacagtTCTGCTGGTGCTCTCATCTGCAAACTGTGGATTATCAAGACCGAAGAGATGCTCGTAGAGAAAGGCTTGAAGCATGGCTGGGATGGGGACTTTGCGTACATGGCATGTGTACATGATGAAATCCAAGTAGGCTGCCGTACCGAATAGATTGCTCAGGTGGTCATTGAGACCGCACAAGAAGCGATGCGCTGGGTTGGAGACCACTGGAACTTCCGGTGTCTTCTGGATACCGAAGGTAAGATGGGTCCTAATTGGGTGATTTGCCACTGATACAGG                                                                                                                                                                                                                                                                                                                  <  1:524197/276‑1`  
`cgtTGCCTTGAATACCCTACTGCAATCTGCTGGTGCTCTCATCTGCAAACTGTGGATTATCTAGACCGACGAGATGCTCGTCGAGAAAGGCTTGAAGCATGGCGGGGATGGGGACTTTGCGTAACTGGCATGGGTACATGATGTAATCCAAGTAGGCTGTCTTAACCAAGAGATTGCTCAGGTGGTCATTGAGACCGCACTAGACGCGATGCGCTGGGTTGGAGCCCACTGGATCTTGCGGGGTCTTCTGGATACCGAAGGTAAGATGGGTCGTAATTGGGCGATTTGCCACTGATACAGG                                                                                                                                                                                                                                                                                                                  >  1:524049/4‑301`  
`CGCTGCCTTGAATACCCTTCTGCAATCTGCTGGTGCTCTCATCTGCAAACTGTGGATTATCAAGACCGAAGAGATGCTCGTAGAGAAAGGCTTGAAGCATGGCTGGGATGGTGACTTTGCGTACATGGCATGGGTACATGATGAAATCCAAGTAGGCTGCCGTACCGAAGAGATTGCTCAGGTGGTCATTGAGACCGCACAAGAAGCGATGCGCTGGGTTGGAGACCACTGGAACTTCCGGTGTCTTCTGGATACCGAAGGTAAGATGGGTCCTAATTGGGCGATTTGCCACTGATAAAGG                                                                                                                                                                                                                                                                                                                  <  1:465419/301‑1`  
 `gtgtataagagacagCTACTGCAATCTGCTGGTGCTCTCATCTGCAAACTGTGGATTATCAAGACCGAAGAGATGCTCGTAGAGAAATGCTTGAAGCATGGCTGTGATGGGGACTTTGCGTACATGGCATGGGTACATGATGAAATCCAAGTAGGCTGCCGTACCGAAGAGATTGCTCAGGTGGTCATTGAGACCGCACAAGAAGCGATGCGCTTGGTTTGAGACCACTGGAACTTCCGGTGTCTTCTGGATACCGAAGGTAAGATTGGTCCTAATTGGGCGATTTGCCACTGATACAGGA                                                                                                                                                                                                                                                                                                                 <  1:797373/286‑1`  
 `GCTGCCTTGAATACCCTACTGCAATCGGGTGGTGCTCTCAGCTGCAAACTGTGGATTATCAAGACCGAAGAGATGCTCGTAGAGAAAGGCTTGAAGCATGGCTGGGATGGGGACTTGGCGTACATGGCATGGGTACATGATGAAATCCAAGTAGGCTGCCGTACCGAAGAGATTGCTCAGGTGGTCATTGAGACCGCACAAGAAGCGATGCGCTGGGTTGGAGACCACTGGAACTTCCGGTGTCTTCTGGATACCGAAGGTAAGATGGGTCCTAATTGGGCGATTTGCCACTGATACAGGA                                                                                                                                                                                                                                                                                                                 <  1:194108/301‑1`  
  `tataagagacagCCCTACTGCAATCTGCTGGTGCTCTCATCTGCAAACTGTGGATTATCAAGACCGAAGAGATGCTCGTAGAGAAAGGCTTGAAGCATGGCTGGGATGGGGACTTTGCGTACATGGCATGGGTACATGATGAAATCCAAGTAGGCTGCCGTACCGAAGAGATTGCTCAGGTGGTCATTGAGACCGCACAAGAAGCGATGCGCTGGGTTGGAGACCACTGGAACTTCCGGTGTCTTCTGGATACCGAAGGTAAGATGGGTCCTAATTGGGCGATTTGCCACTGATACAGGAG                                                                                                                                                                                                                                                                                                                <  1:567927/289‑1`  
  `CTGCCTTGAATACCCTACTGCAATCTGCTGGTGATCTCATCTGCAAACTGTGGATTATCAAGACCGAAGAGATGCTCGTAGAGAAAGGCTTGAAGCATGGCTGGGATGGGGACTTTGCGTACATGGCATGGGTACATGATGAAATCCAAGTAGGCTGCCGTACCGAAGAGATTGCTCAGGTGGTCATTGAGACCGCACAAGAAGCGATGCGCTGGGTTGGAGACCACTGGAACTTCCGGTGTCTTCTGGATACCGAAGGTAAGATGGGTCCTAATTGGGCGATTTGCCACTGATACAGGAG                                                                                                                                                                                                                                                                                                                <  1:17536/301‑1`  
    `GCCTTGAATACCCTACTGCAATCTGCTGGTGCTCTCATCTGCAAACTGTGGATTATCAAGACCGAAGAGATGCTCGTAGAGAAAGGCTTGAAGCATGGCTGGGATGGGGACTTTGCGTACATGGCATGGGTACATGATGAAATCCAAGTAGGCTGCCGTACCGAATAGATTGCTCAGGTGGTCATTGAGACCGCACAAGAAGCGATGCGCTGGGTTGGAGACCACTTGAACTTCCGGTGTCTTCTGGCTACCGAAGGTAAGATTCGTCCTAATTGGGCGATTTGCCACTGATACAGGAGGg                                                                                                                                                                                                                                                                                                              >  1:428742/1‑300`  
    `GCCTTGAATACCCTACTGCAATCTGCTGGTGCTCTCATCTGCAAACTGTGGATTATCAAGACCGAAGAGATGCTCGTAGAGAAAGGCTTGAAGCATGGCTGGGATGGGGACTTTGCGTACATGGCATGGGTACATGATGAAATCCAAGTAGGCTGCCGTACCGAAGAGATTGCTCAGGTGGTCATTGAGACCGCACAAGAAGCGATGCGCTGGGTTGGAGACCACTGGAACTTCCGGTGTCTTCTGGATACCGAAGGTAAGATGGGTCCTAATTGGGCGATTTGCCACTGATACAGGctgt                                                                                                                                                                                                                                                                                                              >  1:626370/1‑297`  
    `GCCTTGAATACCCTACTGCAATCTGCTGGTGCTCTCATCTGCAAACTGTGGATTATCAAGACCGAAGAGATGCTCGTAGAGAAAGGCTTGAAGCATGGCTGGGATGGGGACTTTGCGTACATGGCATGGGTACATGATGAAATCCAAGTAGGCTGCCGTACCGAAGAGATTGCTCAGGTGGTCATTGAGACCGCACAAGAAGCGATGCGCTGGGTTGGAGACCACTGGAACTTCCGGTGTCTTCTGGATACCGAAGGTAAGATGGGTCCTAATTGGGCGATTTGCCACTGATACAGGAGct                                                                                                                                                                                                                                                                                                              >  1:181573/1‑299`  
    `GCCTTGAATACCCTACTGCAATCTGCTGGTGCTCTCATCTGCAAACTGTGGATTATCAAGACCGAAGAGATGCTCGTAGAGAAAGGCTTAAAGCATGGCTGGGATGGGGACTTTGCGTACATGGCATGGGTACATGATGAAATCCAAGTAGGCTGCCGTACCGAAGAGATTGCTCAGGTGGTCATTGAGACCGCACAAGAAGCGATGCGCTGGGTTGGAGACCACTGGAACTTCCGGTGTCTTCTGGATACCGAAGGTAAGATGGGTCCTAATTGGGCGATTTGCCACTGAGACAGGAGGg                                                                                                                                                                                                                                                                                                              >  1:583542/1‑300`  
      `CTTGAATACCCTACTGCAATCTGCTTGTGCTCTCATCTGCAAACTGTGGATTATCAAGACCGAAGAGATGCTCGTAGAGAAAGGCTTGAAGCATGGCTGGGATGGGGACTTTGCGTACATGGCATGGGTACATGATGAAATCCAAGTAGGCTGCCGTACCGAAGAGATTGCTCAGGTGGTCATTGAGACCGCACAAGAAGCGATGCGCTGGGTTGGAGACCACTGGAACTTCCGGTTTCTTCTGGATACCGAAGGTCAGATGGGTCCTAATTGGGCGATTTTCCACTGCTACAGGAGGata                                                                                                                                                                                                                                                                                                            >  1:193118/1‑298`  
      `CTTGAATACCCTACTGCAATCTGCTGGTGCTCTCATCTGCAAACTGTGGATTATCAAGACCGAAGAGATGCTCGTAGAGAAAGGCTTGAAGCATGGCTGGGATGGGGACTTTGCGTACATGGCATGGGTACATGATGAAATCCAAGTAGGCTGCCGTACCGAAGAGATTGCTCAGGTGGTCATTGAGACCGCACAAGAAGCGATGCGCTGGGTTGGAGACCACTGGAACTTCCGGTGTCTTCTGGATACCGAAGGTAAGATGGGTCCTAATTGGGCGATTTGCCACTGATACAGGActgtc                                                                                                                                                                                                                                                                                                            >  1:744093/1‑296`  
      `CTTGAATACCCTACTGCAATCTGCTGGTGCTCTCATCTGCAAACTGTGGATTATCAAGACCGAAGAGATGCTCGTAGAGAAAGGCTTGAAGCATAGCTGGGATGGTGACTTTGCGTACATGGCATGGGTACATGATGAAATCCAAGTAGGCTTCCGTACCGAAGAGATTGCTCAGGTGGTCATTGAGACCGCCCAAGAAGCGATGCGCTGGGTTTGAGACCACTGGAACTTCCTGTGTCTTCTGGATACCGAAGGTAAGATGGGTCCTAATTGGGCGATTTGCCCCTGATACATGAGGata                                                                                                                                                                                                                                                                                                            >  1:170522/1‑298`  
         `GAATACCCTACTGCAATCTGCTGGTGCTCTCATCTGCAAACTGTGGATTATCAAGACCGAAGAGATGCTCGTAGAGAAAGGCTTGAAGCATGGCTGGGATGGGGACTTTGCGTACATGGCATGGGTACATGATGAAATCCAAGTAGGCTGCCGTACCGAAGAGATTGCTCAGGTGGTCATTGAGACCGCACAAGAAGCGATGCGCTGGGTTGGAGACCACTGGAACTTCCAGTGTCTTCTGGATACCGAAGGTAAGATGGGTCCTAATTGGGCGATTTGCCACTGATACAGGctgtctctt                                                                                                                                                                                                                                                                                                         >  1:308386/1‑292`  
           `ATACCCTACTGCAATCTGCTGGTGCTCTCATCTGCAAACTGTGGATTATCAAGACCGAAGAGATGCTCGTAGAGAAAGGCTTGAAGCATGGCTGGGATGGGGACTTTGCGTACATGGCATGGGTACATGATGAAATCCAAGTAGTCTGCCGTACCGAAGAGATTGCTCAGGTGGTCATTGAGACCGCACAAGAAGCGATGCGCTGGGTTGGAGACCACTGGAACTTCCGGTGTCTTCTGGATACCGAAGGTAAGATGGGTCCTAATTGGGCGATTTGCCACTGATACAGGAGCTGTctctt                                                                                                                                                                                                                                                                                                       >  1:597176/1‑296`  
           `ATACCCTACTGCAATCTGCTGGTGCTCTCATCTGCAAACTGTGGATTATCAAGACCGAAGAGATGCTCGTAGAGAAAGGCTTGAAGCATGGCTGGGATGGGGACTTTGCGTACATGGCATGGGTACATGATGAAATCCAAGTAGGCTGCCGTACCGAAGAGATTGCTCAGGTGGTCATTGAGACCGCACAAGAAGCGATGCGCTTGGTTGGAGACCACTGGAACTTCCGGTGTCTTCTGGATACCGAAGGTAAGATGGGTCCTAATTGGGCGATTTGCCACTGATACAGGAGGTGTTCTGA                                                                                                                                                                                                                                                                                                       <  1:467363/301‑1`  
              `CCCTACTGCAATCTGCTGGTGCTCTCATCTGCAAACTGTGGATTATCAAGACCGAAGAGATGCTCGTAGAGAAATGCTTGAAGCATGGCTGGGATGGGGACTTTGCGTACATGGCATGGGTACATGATGAAATCCAAGTAGGCTGCCGTACCGAAGAGATTGCTCAGGTGGTCATTGAGACTGCACAAGAAGCGATGCGCTGGGTTGGAGACCACTGGAACTTCCGGTGTCTTCTGGATACTGAAGGTAAGATGGGTCCTAATTGGGCGATTTGCCACTGATACAGGAGCTGTctcttata                                                                                                                                                                                                                                                                                                    >  1:368811/1‑293`  
              `CCCTACTGCAATCTGCTGGTGCTCTCATCTGCAAACTGTGGATTATCAAGACCGAAGAGATGCTCGTAGAGAAAGGCTTGAAGCATGGCTGGGATGGGGACTTTGCGTACATGGCATGGGTACATGATGAAATCCAAGTAGGCTGCCGTACCGAAGAGATTGCTCAGGTGGTCATTGAGACCGCACAAGAAGCGATGCGCTGGGTTGGAGACCACTGGAACTTCCGGTGTCTTCTGGATACCGAAGGTAAGATGGGTCCTAATTGGGCGATTTGCCACTGATACAGGAGCTGTctcttata                                                                                                                                                                                                                                                                                                    >  1:225542/1‑293`  
               `CCTACTGCAATCTGCTGGTGCTCTCATCTGCAAACTGTGGATTATCAAGACCGAAGAGATGCTCGTAGAGAAAGGCTTGAAGCATGGCTGGTATGGGGACTTTGCGTACATGGCATGGGTACATGATGAAATCCAAGTAGGCTGCCGTACCGAAGAGATTGCTCAGGTGGTCATTGAGACTGCACAAGAAGCGATGCGCTGGGTTGGAGACCACTGGAACTTCCGGTGTCTTCTGGATACCGAAGGTAAGATGGGTCCTAATTGGGCGATTTGCCACTGATACAGGAGGTGTTCTGA‑TGTC                                                                                                                                                                                                                                                                                                  <  1:547684/301‑1`  
                     `GCAATCTGCTGGTGCTCTCATCTGCAAACTGTGGATTATCAAGACCGAAGAGATGCTCGTAGAGAAAGGCTTGAAGCATGGCTGGGATGGGGACTTTGCGTACATGGCATGGGTACATGATGAAATCCAAGTAGGCTTCCGTACCGAAGAGATTGCTCATGTGGTCATTGAGACCGCACAAGAAGCGATGCGCTGGGTTGGAGACCACTGGAACTTCCGGTGTCTTCTGGATACCGAAGGTAAGATGGGTCCTAATTGGGCTATTTGCCACTGATACAGGAGCTGTctcttatacacatct                                                                                                                                                                                                                                                                                             >  1:624162/1‑286`  
                          `CTGCTGGTGCTCTCATCTGCAAACTGTGGATTATCAAGACCGAAGAGATGCTCGTAGAGAAAGGCTTGAAGCATGGCTGGGATGGGGACTTTGCGTACATGGCATGGGTACATGATGAAATCCAAGTAGGCTGCCGTACCGAAGAGATTGCTCAGGTGGTCATTGAGACCGCACAAGAAGCGATGCGCTGGGTTGGAGACCACTGGAACTTCCGGTGTCTTCTGGATACCGAAGGTAAGATGGGTCCTAATTGGGCGATTTGCCACTGATACAGGAGGTGTTCTGACTGTCTcttatacac                                                                                                                                                                                                                                                                                        >  1:77027/1‑292`  
                                                   `GTGGATTATCAAGACCGAAGAGATGCTCGTAGAGAAAGGCTTGAAGCATGGCTGGGATGGGGACTTTGCGTACATGGCATGGGTACATGATGAAATCCAAGTAGGCTGCCGTACCGAAGAGATTGCTCAGGTGGTCATTGAGACCGCACAAGAAGCGCTGCGCTGGGTTGGAGACCACTGGAACTTCCGGTGTCTTCTGGATACCGAAGGTAAGATGGGTCCTAATTGGGCGATTTGCCACTGATACAGGAGGTGTTCTGA‑TGTCTGACTACCTGAAAGTGCTGCAAGCAATCAAAAGTTt                                                                                                                                                                                                                                                              >  1:675373/1‑300`  
                                                   `GTGGATTATCAAGACCGAAGAGATGCTCGTAGAGAAAGGCTTGAAGCATGGATGGGATGGGGACTTTGCGTACATGGCATGGGTACATGATGAAATCCAAGTAGGCTGCCGTACCGAAGAGATTGCTCAGGTGGTCATTGAGACCGCACAAGAAGCGATGCGCTGGGTTGGAGACCACTGTAACTTCCGGTGTCTTCTGGATACCGAAGGTAAGATGGGTCCTAATTGGGCGATTTGCCACTGATACAGGAGGTGGTCTGA‑TGTCTGACTAACTGAAAGTGCTGCAAGCAATCAAAAGTTt                                                                                                                                                                                                                                                              >  1:675389/1‑300`  
                                                     `GGATTATCAAGACCGAAGAGATGCTCGTAGAGAAAGGCTTGAAGCATGGCTGGGATGGGGACTTTGCGTACATGACATGGGTACATGATGAAATCAAAGTAGGCTGCCGTACCGAAGAGATTGCTCAGGTGGTCATTGAGACCGCACAAGAAGCGATGCGCTGGGTTGGAGACCACTGGAACTTCCGGTGTCTTCTGGATACCGAAGGTAAGATGGGTCCTAATTGGGCGATTTGCCACTGATACAGGAGGTGTTCTGA‑TGTCTGACTACCTGAAAGTGCTGCAAGCAATCAAAAGTTGCC                                                                                                                                                                                                                                                            >  1:215810/1‑301`  
                                                          `ATCAAGACCGAAGAGATGCTCGTAGAGAAAGGCTTGAAGCATGGCTGGGATGGGGACTTTGCGTACATGGCATGGGTACATGATGAAATCCAAGTAGGCTGCAGTACCAAAGAGATTGCTCAGGTGGTCATTGAGACCGCACAAGAAGCGATGCGCTGGGTTGGAGACCACTGGAACTTCCGGTGTCTTCTGGATACCGAAGGTAAGATGGGTCCTAATTGGGCGATTTGCCACTGATACAGGAGGTGTTCTGA‑TGTCTGACTACCTGAAAGTGCTGCAAGCAATCAAAAGTTGCCCTAAG                                                                                                                                                                                                                                                       >  1:396512/1‑301`  
                                                                   `GAAGAGATGCTCGTAGAGAAAGGCTTGAAGCATGGCTGGGATGGGGACTTTGCGTACATGGCATGGGTACATGATGAAATCCAAGTAGGCTGCCGTACCGAAGAGATTGCTCAGGTGGTCATTGAGACCGCACAAGAAGCGATGCGCTGGGTTGGAGACCACTGGAACTTCCGGTGTCTTCTGGATACCGAAGGTAAGATGGGTCCTAATTGGGCGATTTGCCACTGATACAGGAGGTGTTCTGA‑TGTCTGACTACCTGAAAGTGCTGCAAGCAATCAAAAGTTGCCCTAAGACTTTCCAG                                                                                                                                                                                                                                              >  1:572114/1‑301`  
                                                                     `AGAGATGCTCGTAGAGAAAGGCTTGAAGCATGGCTGGGATGGGGACTTTGCGTACATGGCATGGGTACATGATGAAATCCAAGTAGGCTGCCGTACCGAAGAGATTGCTCAGGTGGTAATTGAGACCGCACAAGAAGCGATGCGCTGGGTTGGAGACCACTGGAACTTCCTGTGTCTTCTGGATACCGAAGGTAAGATGGGTCCTAATTGGGCGATTTGCCACTGATACAGGAGGTGTTCTGA‑TGTCTGACTACCTGAAAGTGCTGCAAGCAATCAAAAGTTGCCCTAAGACTTTCAAGTC                                                                                                                                                                                                                                            <  1:474305/301‑1`  
                                                                      `ctcggagatgtgtataagagacaGAAGCATTGCTGGGATGGGGACTTTGCGTACATGGCATGGGTACATGATGAAATCCAAGTAGGCTGCCGTACCGAAGAGATTGCTCAGGTGGTCATTGAGACCGCACAAGAAGCGATGCGCTGGGTTGGAGACCACTGGAACTTCCGGTGTCTTCTGGATACCGAAGGTAAGATGGGTCCTAATTGGGCGATTTGCCACTGATACAGGAGGTGTTCTGA‑TGTCTGACTACCTGAAAGTGCTGCAAGCAATCAAAAGTTGCCCTAAGACTTTCCAGTCC                                                                                                                                                                                                                                           <  1:523131/278‑1`  
                                                                                               `AGCATGGCTGGGATGGGGACTTTGCGTACATGGCATGGGTACATGATGAAATCCAAGTAGGCTGCCGTACCGAAGAGATTGCTCAGGTGGTCATTGAGACCGCACAAGAAGCGATGCGCTGGGTTGGAGACCACTGGAACTTCCGGTGTCTTCTGGATACCGAAGGTAAGATGGGTCCTAATTGGGCGATTTGCCACTGATACAGGAGGTGTTCTGA‑TGTCTGACTACCTGAAAGTGCTGCAAGCAATCAAAAGTTGCCCTAAGACTTTCCAGTCCAACTATGTACGGAACAATGCGAGCC                                                                                                                                                                                                                  <  1:146444/301‑1`  
                                                                                                   `TGGCTGGGATGGGGACTTTGCGTACATGGCATGGGTACATGATGAAATCCAAGTAGGCTGCCGTACCGAAGAGATTGCTCAGGTGGTCATTGAGACCGCACAAGAAGCGATGCGCTGGGTTGGAGACCACTGGAACTTCCGGTGTCTTCTGGATACCGAAGGTAAGATGGGTCCTAATTGGGCGATTTGCCACTGATACAGGAGGTGTTCTGA‑TGTCTGACTACCTGAAAGTGCTGCAAGCAATCAAAAGTTGCCCTAAGACTTTCCAGTCCAACTATGTACGGActgtctcttatacaca                                                                                                                                                                                                              >  1:240968/1‑285`  
                                                                                                      `ctcggagatgtgtataagagACAGGGCATGGGTACATGATGAAATACAAGTAGGCTGCCGTACCGAAGAGATTGCTCAGGTGGTCATTGAGACCGCACAAGAAGCGATGCGCTGGGTTGGAGACCACTGGAACTTCCGGTGTCTTCTGGATACCGAAGGTAAGATGGGTCCTAATTGGGAGATTTGCCACTGATACAGGAGGTGTTCTGA‑TGTCTGACTACCTGAAAGTGCTGCAAGCAATCAAAAGTTGCCCTAAGACTTTCCAGTCCAACTATGTACGGAACAATGCGAGCCTCGTAGC                                                                                                                                                                                                           <  1:192630/281‑1`  
                                                                                                                      `GCGTACATGGCATGGGTACATGATGAAATCCAAGTAGGCTGCCGTACCGAAGAGATTGCTCAGGTGGTCATTGAGACCGCACAAGAAGCGATGCGCTGGGTTGGATACCACTGGAACTTCCGGTGTCTTCTGGATACCGAAGGTAAGATGGGTCCTAATTGGGCGATTTGCCACTGATACAGGAGGTGTTCTGA‑TGTCTGACTACCTGAAAGTGCTGCAAGCAATCAAAAGTTGCCCTAAGACTTTCCAGTCCAACTATGTACGGCACAATGCGAGCCTCGTAGTGGAGGCCGCTTCCCGT                                                                                                                                                                                           >  1:119840/1‑301`  
                                                                                                                                                `AATCCAAGTAGGCTGCCGTACCGAAGAGATTGCTCAGGTGGTCATTGAGACCGCACAAGAAGCGATGCGCTGGGTTGGAGACCACTGGAACTTCCGGTGTCTTCTAGATACCGAAGGTAAGATGGGTCCTAATTGGGCGATTTGCCACTGATACAGGAGGTGTTCTGA‑TGTCTGACTACCTGAAAGTGCTGCAAGCAATCAAAAGTTGCCCTAAGACTTTCCAGTCCAACTATGTACGGAACAATGCGAGCCTCGTAGCGGAGGCCGCTTCCCGTGGTCACATCTCGTGCCTGACTACTAt                                                                                                                                                                 >  1:534695/1‑300`  
                                                                                                                                                                    `CCGAAGAGATTGCTCAGGTGGTCATTGAGACCGCACAAGAAGCGATGCGCTGGGTTGGAGACCACTGGAACTTCCGGTGTCTTCTGGAGACCGAAGGTAAGATGGGTCCTAATTGGGCGATTTGCCACTGATACAGGAGGTGTTCTGA‑TGTCTGACTACCTGAAAGTGCTGCAAGCAATCAAAAGTTGACCTAAGAATTTCCAGTACAACTATGTACGGAAAAATGCGAGCCTCGTAGCGGAGGCCGCTTCCCGTGGTCACATCTCGTGCCTGACTACTAGTGGACGGAACGGTGGCGGTT                                                                                                                                             >  1:432860/1‑301`  
                                                                                                                                                                                       `GGTCATTGAGACCGCACAAGAAGCGATGCGCTGGGTTGGAGACCACTGGAACTTCCGGTGTCTTCTGGATACCGAAGGTAAGATGGGTCCTAATTGGGCGATTTGCCACTGATACAGGAGGTGTTCTGA‑TGTCTGACTACCTGAAAGTGCTGCAAGCAATCAAAAGTTGCCCTAAGACTTTCCAGTCCAACTATGTACGGAACAATGCGAGCCTCGTAGCGGAGGCCGCTTCCCGTGGTCACATCTCGTGCCTGACTACTAGTGGACGTAACGGTGGCGCTTGGGAAATCACTGCTTCCGG                                                                                                                          <  1:517973/301‑1`  
                                                                                                                                                                                          `CATTGAGACCGCACAAGAAGCGATGCGCTGGGTTGGAGACCACTGGAACTTCCGGTGTCTTCTGGATACCGAAGGTAAGATGGGTCCTAATTGGGCGATTTGCCACTGATACAGGAGGTGTTCTGA‑TGTCTGACTACCTGAAAGTGCTGCAAGCAATCAAAAGTTGCCCTAAGACTTTCCAGTCCAACTATGTACGGAACAATGCGAGCCTCGTAGCGGAGGCCGCTTACCGTGGTCACATCTCGTGCCTGACTACTAGTGGACGTAACGGTGGCtgtctcttatacacatctccgagccc                                                                                                                       >  1:62170/1‑275`  
                                                                                                                                                                                                     `CACAAGAAGCGATGCGCTGGGTTGGAGACCACTGGAACTTCCGGTGTCTTCTGGATACCGAAGGTAAGATGGGTCCTAATTGGGCGATTTGTCAATGATACAGGCGGTGTTCTGA‑TGTCTGACTACCTGACAGTGCTGCAAGCAATCAAAAGTTGCCCTCAGACTTTCTAGTTCAACTATGTACGGAACAATGCGAGCCTCGTAGCGTAGGCCGCTTCCCGTGGTTACATCTCGTGCCTGACTACTAGTTGACGTCACAGTGGCGCTTGTGAAATCACTGCTTCAGGTACTCGCTTTCTGA                                                                                                            >  1:406438/1‑301`  
                                                                                                                                                                                                       `CAAGAAGCGATGCGCTGGGTTGGAGACCACTGTAACTTCCGGTGTCTTCTGGATACCGAAGGTAAGATGGGTCCTAATTGGGCGATTTGCCACTTATACACGAGGTGTTCTGA‑TGTCTGACTACCTGAAAGTGCTGCAAGCAATCAAAAGTTGCCCTAAGACTTTCCAGTCCAACTATGTACGGAACAATGCGAGCCTCGTAGCGGAGGCCGCTTCCCGTGGTCACATCTCGTGCCTGACTACTAGTGGACGTAATGGTGGCGCTTGGGAAATCACTGCTTCCGGTACTCGCTTTCTGAAA                                                                                                          >  1:15869/1‑301`  
                                                                                                                                                                                                                         `agagacagCCACTGGAACTTCCGGTGTCTTCTGGATACCGAAGGTAAGATGGGTCCTAATTGGGCGATTTGCCACTGATACAGGAGGTGTTCTGA‑TGTCTGACTACCTGAAAGTGCTGCAAGCAATCAAAAGTTGCCCTAAGACTTTCCAGTCCAACTATGTACGGAACAATGCGAGCCTCGTAGCAGAGGCCGCTTCCCGTGGTCACATCTCGTGCCTGACTACTAGTGGACGTAACGGTGGCGCTTGGGAAATCACTGCTTCCGGTACTCGCTTTCTGAAACGAATGGGAGGATGTGTC                                                                                        <  1:610034/293‑1`  
                                                                                                                                                                                                                               `ggctcggagatgtgtataagagacagGGATACCGAAGGTAAGATGGGTCCTAATTGGGCGAGTTGCCACTGATACAGGAGGTGTTCTGA‑TGTCTGACTACCTGAAAGTGCTGCAAGCAATCAAAAGTTGCCCTAAGACTTTCCAGTCCAACTATGTACGGAACAATGCGAGCCTCGTAGCGGAGGCCGCTTCCCGTGGTCACATCTCGTGCCTGACTACTAGTGGACGTAACGGTGGCGCTTGGGAAATCACTGCTTCCGGTACTCGCTTTCTGAAACGAATGGGAGTATGTGTCTAATGT                                                                                  <  1:376522/275‑1`  
                                                                                                                                                                                                                               `ggagatgtgtataagagacagCTTCTGGATACCGAAGGTAAGATGGGTCCTAATTGGGCGATTTGCCACTGATACAGGAGGTGTTCTGA‑TGTCTGACTACCTGAAAGTGCTGCAAGCAATCAAAAGTTGCCCTAAGACTTTCCAGTCAAACTATGTACGGAACAATGCGAGCCTCGTAGCGGAGGCCGCTTCCCGTGGTCACATCTCGTGCCTGACTACTAGTGGACGTAACGGTGGCGCTTGGGAAATCACTGCTTCCGGTACTCGCTTTCTGAAACGAATGGGAGGATGTGTCTAATGT                                                                                  <  1:617575/280‑1`  
                                                                                                                                                                                                                                        `agCTTCCGGTGTCTTCTGGATACCGAAGGTAAGATTGGTCCTAATTGGGCGATTTGCCACTGATACAGGAGGTGTTCTGA‑TGTCTGACTACCTGAAAGTGCTGCAAGCAATCAAAAGTTGCCCTAAGACTTTCCAGTCCAACTATGTACGGAACAATGCGAGCCTCGTAGCGGAGGCCGCTTCCCGTGGTCACATCTCGTGCCTGACTACTAGTGGACGTAACGGTGGCGCTTGGGAAATCACTGCTTCCGGTACTCGCTTTCTGAAACGAATGGGAGGATGTGTCTAATGTCTCGTGACC                                                                         <  1:573519/299‑1`  
                                                                                                                                                                                                                                                  `GTCTTCTGGATACCGAAGGTAAGATGGGTCCTAATTGGGCGATTTGCCACTGATACAGGAGGTGTTCTGA‑TGTCTGACTACCTGAAAGTGCTGCAAGCAATCAAAAGTTGCCCTAAGACTTTCCAGTCCAACTATGTACGGAACAATGCGAGCCTCGTAGCGGAGGCCGCTTCCAGTGGTCACATCTCGTGCCTGACTACTAGTGGACGTAACGGTGGCGCTTGGGAAATCACTGCTTCCGGTACTCGCTTTCTGAAACGAATGGGAGGATGGGTCTAATGTCTgtctcttatacacatct                                                               >  1:502969/1‑284`  
                                                                                                                                                                                                                                                        `TGGATACCGAAGGTAAGATGGGTCCTAATTGGGCGATTTGCCACTGATACATGAGGTGTTGTGA‑TGTCTGACTACCTGAAAGTGCTGCAAGCAATCAAAAGTTGCCCTAAGACTTTCCAGTCCAACTATGTACGGAAAAAGGCGAGCCTCGTAGCGGAGGCCGCTTCCCGTGGTCACATCTCGTGCCTGACTACTAGTGGACGTAACGGTGGCGCTTGGGAAATCACTGCTTCCGGTACTCGCTTTCTGAAACGAATGGGAGGATGTGTCTAATGTCTCGTGACCTTGTGACTATTCCACG                                                         <  1:759923/301‑1`  
                                                                                                                                                                                                                                                                        `GATGGGTCCTAATTGGGCGATTTGCCACTGATACAGGAGGTGTTCTGA‑TGTCTGACTACCTGAAAGTGCTGCAAGCAATCAAAAGTTGCCCTAAGACTTTCCAGTCCAACTATGTACGGAACAATGCTAGCCTCGTAGCGGAGGTCGCTTCCCGTGGTCACATCTCGTGCCTGACTACTAGTGGACGTAACGGTGGCGCTTGTGAAATCACTGCTTCCGGTACTCGCTTTCTGAAACGAATGGGAGGATGTGTCTAATGTCTCGTGACCTTGTGACTATTCCACGCGActgtctcttataa                                         >  1:607556/1‑288`  
                                                                                                                                                                                                                                                                            `GGTCCTAATTGGGCGATTTGCCACTGATACAGGAGGTGTTCTGA‑TGTCTGACTACCTGAAAGTGCTGCAAGCAATCAAAAGTTGCCCTAAGACTTTCCAGTCCAACTATGTACGGAACAATGCGAGCCTCGTAGCGGAGGCAGCTTCCCGTGGTCACATCTCGTGCCTGACTACTAGTGGACGTAACGGTGGCGCTTGGGAAATCACTGCTTCCGGTACTCGCTTTCTGAAACGAATGGGAGGATGTGTCTAATGTCTCGTGACCTTGTGACTATTCAACGCGATGTGTGGAACGATATAa                                     >  1:400903/1‑300`  
                                                                                                                                                                                                                                                                             `GTCCTAATTGGGCGATTTGCCACTGATACAGGAGGTGTTCTGA‑TGTCTGACTACCTGAAAGTGCTGCAAGCAATCAAAAGTTGCCCTAAGACTTTCCAGTCCAACTATGTACGGAACAATGCGAGCCTCGTAGCGGATGCCGCTTCCCGTGGTCACATCTCGTGCCTGACTACTAGTGGACGTAATGGTGGCGCTTGGGAAATCACTGCTTCCGGTACTCGCTTTCTGAAACGAATGGGAGGATGTGTCTAATGTCTCGTGACCTTGTGACTATTCCACTCGATGTGTGGAACGATATACc                                    >  1:573536/1‑300`  
                                                                                                                                                                                                                                                                                `cggagatgtgtataagagacaGATACAGGAGGTGTTCTGA‑TGTCTGACTACCTGAAAGTGCTGCAAGCAATCAAAAGTTGCCCTAAGACTTTCCAGTCCAACTATGTACGGAACAATGCGAGCCTCGTAGCGGAGGCCGCTTCCCGTGGTCACATCTCGTGCCTGACTACTAGTGGACGTAACGGTGGCGCTTGGGAAATCACTTCTTCCGGTACTCGCTTTCTGAAACGAATGGGAGGATGTGTCTAATGTCTCGTGACCTTGTGACTATTCCACGCGATGTGTGGAACGATATACAGGG                                 <  1:90784/280‑1`  
                                                                                                                                                                                                                                                                                 `cggagatgtgtataagagacagTACAGGAGGTGTTCTGA‑TGTCTGACTACCCGAAAGTGATGCAAGCAATCAAAAGTTGCCCTAAGACTTTCCAGTCCAACTATGTACGGAACAATGCGAGCCTCGTAGCGGAGGCCGCTTCCCGTGGTAACATCTCGTGCCTGACTACTAGTGGACGTAACGGTGGCGCTTGGGAAATCACTGCTTCCGGTACTCGCTTTCTGAAACGAATGGGAGGATGTGTCTAATGTCTCGTGACCTTGTGACTATTCCACGCGATGTGTGGAACGATATACAGGGC                                <  1:201671/279‑1`  
                                                                                                                                                                                                                                                                                 `TAATTGGGCGATTTGCCACTGATACAGGAGGTGTTCTGA‑TGTCTGACTACCTGAAAGTGCTGCAAGCAATCAAAAGTTGCCCTAAGACTTTCCAGTCCAACTATGTACGGAACAATGCGAGCCTCGTAGCGGAGGCCGCTTCCAGTGGTCACATCTCGTGCCTGACTACTAGTGGACGTAACGGTGGCGCTTGGGAAATCACTGCTTCCGGTACTCGCTTTCTGAAACGAATGGGAGGATGTGTCTAATGTCTCGTGACCTTGTGACTATTCCACGCGATGTGTGGAACGATATACAGGGC                                <  1:677560/301‑1`  
                                                                                                                                                                                                                                                                                      `ctcggagatgtgtataagagacagGGTGTTCTGA‑TGTCTGACTACCTGAAAGTGCTGCAAGCAATCAAAAGTTGCCCTAAGACTTTCCAGTCCAACTATGTACGGAACAATGCGAGCCTCGTAGCGGAGGCCGCTTCCCGTGGTCACATCTCGTGCCTGACTACTAGTGGACGTAACGGTGGCGCTTGGGAAATCACTGCTTCCGGTACTCGCTTTCTGAAACGAATGGGAGGATGTGTCTAATGTCTCGTGACCTTGTGACTATTCCACGCGATGTGTGGAACGATATACAGGGCTACAT                           <  1:404843/277‑1`  
                                                                                                                                                                                                                                                                                                    `aGATACAGTAGGTGTTCTGA‑TGTCTGACTACCTGAAAGTGCTGCAAGCAATCAAAAGTTGCCCTAAGACTTTCCAGTCCAACTATGTACGGAACAATGCGAGCCTCGTAGCGGAGGCCGCTTCCCGTGGTCACATCTCGTGCCTGACTACTAGTGGACGTAACGGTGGAGCTTGGGAAATCACTGCTTCCGGTACTCGCTTTCTGAAACGAATGGGAGGATGTGTCTAATGTCTCGTGACCTTGTGACTATTCCACGCGATGTGTGGAACGATATACAGGGCTACATCGACTCTCTGGAAC             <  1:704613/300‑1`  
                                                                                                                                                                                                                                                                                                      `ATACAGGAGGTGTTCTGA‑TGTCTGACTACCTGAAAGTGCTGCAAGCAATCAAAAGTTGCCCTAAGACTTTCCAGTCCAACTATGTACGGAACAATGCGAGCCTCGTAGCGGAGGCCGCTTCCCGTGGTCACATCTCGTGCCTGACTACTAGTTGACGTAACGGTGGCGCTTGGGTAATCACTGCTTCCGGTACTCGCTTTCTGAAACGAATGGGAGGATGTGTCTAATGTCTCGTGACCTTGTGACTATTCCACGCGATGTGTGGAACGATATACAGGGCTACATCtgtctcttatacaca           >  1:300553/1‑286`  
                                                                                                                                                                                                                                                                                                        `ACAGGAGGTGTTCTGA‑TGTCTGACTACCTGAAAGTGCTGCAAGCAATCAAAAGTTGCCCTAAGACTTTCCAGTCCAACTATGTACGGAACAATGCGAGCCTCGTAGCGGAGGCCGCTTCCCGTGGTCACATCTCGTGCCTTACTACTAGTGGACGTAACGGTGGCGCTTGGGAAATCACTGCTTCCGGTACTCGCTTTCTGAAACGAATGGGAGGATGTGTCTAATGTCTCGTGACCTTGTGACTATTCCACGCGATGTGTGGAACGATATACAGGGCTACATCGACTCTCTGGAACCTGt         >  1:408474/1‑300`  
                                                                                                                                                                                                                                                                                                           `GGAGGTGTTCTGA‑TGTCTGACTACCTGAAAGTGCTGCAAGCAATCAAAAGTTGCCCTAAGACTTTCCAGTCCAACTATGTACGGAACAATGCGAGCCTCGTAGCGGAGGCCGCTTCCCGTGGTCACATCTCGTGCCTGACTACTAGTGGACGTAACGGTGGCGCTTGGGAAATCACTGCTTCCGGTACTCGCTTTCTGAAACGAATGGGAGGATGTGTCTAATGTCTCGTGACCTTGTTACTATTCCACGCGATGTGTGGAACGATATACAGGctgtctcttatacacatctccgagccca      >  1:250533/1‑273`  
                                                                                                                                                                                                                                                                                                           `GGAGGTGTTCTGA‑TGTCTGACTACCTGAAAGTGCTGCAAGCAATCAAAAGTTGCCCTAAGACTTTCCAGTCCAACTATGTACGGAACAATGCGAGCCTCGTAGCGGAGGCCGCTTCCCGTGGTCACATCTCGTGCCTGACTACTAGTGGACGTAACGGTGGCGCTTGGGAAATCACTGCTTCCGGTACTCGCTTTCTGAAACGAATGGGAGGATGTGTCTAATGTCTCGTGACCTTGTGACTATTCCACGCGATGTGTGGAACGATATACAGGGCTACATCGACTCTCTGGAACGTGAGAA      >  1:252313/1‑301`  
                                                                                                                                                                                                                                                                                                           `GGAGGTGTTCTGA‑TGTCTGACTACCTGAAAGTGCTGCAAGCAATCAAAAGTTGCCATAAGACTTTCCAGTCCAACTATGTACGGAACAATGCGAGCCTCGTAGCGGAGGCCGCTTCCCGTGGTCACATCTCGTGCCTGACTACTAGTGGACGTAACGGTGGCGCTTGGGAAATCACTGCTTCCGGTACTCGCTTTCTGAAACGAATGGGAGGATGTGTCTAATGTCTAGTGACCTTGTGACTATTCCACGCGATGTGTGGAACGATATACAGGGCTACATCGACTCTCTGGAACCTGtctc      >  1:748267/1‑297`  
                                                                                                                                                                                                                                                                                                            `agGGTGTTCTGA‑TGTCTGACTACCTGAAAGTGCTGCAAGCAATCAAAAGTTGCCCTAAGACTTTCCAGTCCAACTATGTACGGAACAATGCGAGCCTCGTAGCGGAGGCCGCTTCCCGTGGTCACATCTCGTGCCTGACTACTAGTGGACGTAACGGTGGCGCTTGGGAAATCACTGCTTCCGGTACTCGCTTTCTGAAACGAATGGGAGGATGTGTCTAATGTCTCGTGACCTTGTGACTATTCCACTCGATGTGTGGAACGATATACAGGGCTACATCGACTCTCTTGAACGTGAGAAC     <  1:224137/299‑1`  
                                                                                                                                                                                                                                                                                                            `aAGGTGTTCTGA‑TGTCTTACTACCTGAAAGTGCTGCAAGCAATCAAAAGTTGCCCTAAGACTTTCCAGTCCAACTATGTACGGAACAATGCGAGCCTCGTAGCGGAGGCCGCTTCCCGTGGTAACATCTCGTGCCTGACTACTAGTGGACGTAACGGTGGCGCTTGGGAAATCACTGCTTCCGGTACTCGCTTTCTGAAACGAATGGGAGGATGTGTCTAATGTCTCGTGACCTTGTGACTATTCCACGCGATGTGTGGAACGATATACAGGGCTACATCGACTCTCTGGAACGTGAGAAC     <  1:343855/300‑1`  
                                                                                                                                                                                                                                                                                                            `GAGGTGTTCTGA‑TGTCTGACTACCTGAAAGTGCTGCAAGCAATCAAAAGTTGCCCTAAGACTTTCCAGTCCAACTATGTACGGAACAATGCGAGCCTCGTAGCGGAGGCCGCTTCCCGTGGTCACATCTCGTGCATGACTACTAGTGGACGTAACGGTGGAGCTTGGGAAATCACTGCTTCCGGTACTCGCTTTCTGAAACGAATGGGAGGATGTGTCTAATGTCTCGTGACCTTGTGACTATTCCACGCGATGTGTGGAACGATATACAGGGCTACATCGACTCTCTGGAACGTGAGAAC     <  1:757661/301‑1`  
                                                                                                                                                                                                                                                                                                            `GAGGTGTTCTGA‑TGTCTGACTACCTGAAAGTGATGCAAGCAATCAAAAGTTGCCCTAAGACTTTCCAGTCCAACTATGTACGGAACAATGCGAGCCTCGTAGCGGAGGACGCTTCCCGTGGTCACATCTCGTGCCTGACTACTAGTGGACGTAACGGTGGCGCTTGGGAAATCACTGCTTCCGGTACTCGCTTTCTGAAACGAATGGGAGGATGTGTCTAATGTCTCGTGACCTTGTGACTATTCCACGCGATGTGTGGAACGATATACAGGGCTctgtctcttatacacatctccgagcc     >  1:437423/1‑275`  
                                                                                                                                                                                                                                                                                                               `GTGTTCTGA‑TGTCTGACTACCTGAAAGTGCTGCAAGCAATCAAAAGTTGCCCTAAGACTTTCCAGTCCAACTATGTACGGAACAATGCGAGCCTCGTAGCGGAGGCCGCTTCCCGTGGTCACATCTCGTGCCTGACTACTAGTGGACGTAACGGTGGCGCTTGGGAAATCACTGCTTCCGGTACTCGCTTTCTGAAACGAATGGGAGGATGTGTCTAATGTCTCGTGACCTTGTGACTATTCCACGCGATGTGTGGAACGATATACAGGGCTACATCGACTCTATGGAACGTGAGAACGAT  <  1:614376/301‑1`  

                                                                                                                                                                                                                                                                                                                                                                                                                                                                                                                                                                                                                               
`CGCTGCCTTGAATACCCTACTGCAATCTGCTGGTGCTCTCATCTGCAAACTGTGGATTATCAAGACCGAAGAGATGCTCGTAGAGAAAGGCTTGAAGCATGGCTGGGATGGGGACTTTGCGTACATGGCATGGGTACATGATGAAATCCAAGTAGGCTGCCGTACCGAAGAGATTGCTCAGGTGGTCATTGAGACCGCACAAGAAGCGATGCGCTGGGTTGGAGACCACTGGAACTTCCGGTGTCTTCTGGATACCGAAGGTAAGATGGGTCCTAATTGGGCGATTTGCCACTGATACAGGAGG‑‑‑‑‑‑‑‑‑‑‑‑‑‑‑‑‑‑‑‑‑‑‑‑‑‑‑‑‑‑‑‑‑‑‑‑‑‑‑‑‑‑‑‑‑‑‑‑‑‑‑‑‑‑‑‑‑‑‑‑‑‑‑‑‑‑‑‑‑‑‑‑‑‑‑‑‑‑‑‑‑‑‑‑‑‑‑‑‑‑‑‑‑‑‑‑‑‑‑‑‑‑‑‑‑‑‑‑‑‑‑‑‑‑‑‑‑‑‑‑‑‑‑‑‑‑‑‑‑‑‑‑‑‑‑‑‑‑‑‑‑‑‑‑‑‑‑‑‑‑‑‑‑‑‑‑‑‑‑‑‑‑‑‑‑‑‑‑‑‑‑‑‑‑‑‑‑‑‑‑‑‑‑‑‑‑‑‑‑‑‑‑‑‑‑‑‑‑‑‑‑‑‑‑‑‑‑‑‑‑‑‑‑‑‑‑‑‑‑‑‑‑‑‑‑‑‑‑‑‑‑‑‑‑‑‑‑‑‑‑‑‑‑‑‑‑‑‑‑‑‑‑‑‑‑‑‑‑‑‑‑‑‑‑‑‑‑‑‑‑‑‑‑‑‑‑‑‑‑‑‑‑‑‑‑‑‑‑‑‑‑‑‑‑‑‑‑‑‑‑‑  >  NC_001604.1/16173‑16476`  
`‑‑‑‑‑‑‑‑‑‑‑‑‑‑‑‑‑‑‑‑‑‑‑‑‑‑‑‑‑‑‑‑‑‑‑‑‑‑‑‑‑‑‑‑‑‑‑‑‑‑‑‑‑‑‑‑‑‑‑‑‑‑‑‑‑‑‑‑‑‑‑‑‑‑‑‑‑‑‑‑‑‑‑‑‑‑‑‑‑‑‑‑‑‑‑‑‑‑‑‑‑‑‑‑‑‑‑‑‑‑‑‑‑‑‑‑‑‑‑‑‑‑‑‑‑‑‑‑‑‑‑‑‑‑‑‑‑‑‑‑‑‑‑‑‑‑‑‑‑‑‑‑‑‑‑‑‑‑‑‑‑‑‑‑‑‑‑‑‑‑‑‑‑‑‑‑‑‑‑‑‑‑‑‑‑‑‑‑‑‑‑‑‑‑‑‑‑‑‑‑‑‑‑‑‑‑‑‑‑‑‑‑‑‑‑‑‑‑‑‑‑‑‑‑‑‑‑‑‑‑‑‑‑‑‑‑‑‑‑‑‑‑‑‑‑‑‑‑‑‑‑‑‑‑‑‑‑‑‑‑‑‑‑‑‑‑‑‑‑‑‑‑‑‑‑‑‑‑‑‑‑‑‑‑‑‑‑‑‑‑‑‑‑‑‑‑‑‑‑ggaggTGTTCTGA‑TGTCTGACTACCTGAAAGTGCTGCAAGCAATCAAAAGTTGCCCTAAGACTTTCCAGTCCAACTATGTACGGAACAATGCGAGCCTCGTAGCGGAGGCCGCTTCCCGTGGTCACATCTCGTGCCTGACTACTAGTGGACGTAACGGTGGCGCTTGGGAAATCACTGCTTCCGGTACTCGCTTTCTGAAACGAATGGGAGGATGTGTCTAATGTCTCGTGACCTTGTGACTATTCCACGCGATGTGTGGAACGATATACAGGGCTACATCGACTCTCTGGAACGTGAGAACGAT  >  NC_001604.1/17143‑17442`

  

Alignment Legend

Aligned base mismatch/match (shaded by quality score): `ATCG/ATCG` `< 3 ≤` `ATCG/ATCG` `< 15 ≤` `ATCG/ATCG` `< 27 ≤` `ATCG/ATCG` `< 37 ≤` `ATCG/ATCG` `< 38 ≤` `ATCG/ATCG`

Unaligned base: `atcg`    Masked matching base: `atcg`    Alignment gap: `‑`    Deleted base: `‑`

Reads not counted as support for junction

read\_name Not counted due to insufficient overlap past the breakpoint.

read\_name Not counted due to not crossing MOB target site duplication.
